# Supplementary material for: Zn2+ is Essential for Ca2+ Oscillations in Mouse Eggs
Source: bioRxiv. 2023 Aug 29:2023.04.13.536745. Preprint. [Version 3] doi: 10.1101/2023.04.13.536745 (PMC10153198; doi:10.1101/2023.04.13.536745)

bioRxiv preprint doi: https://doi.org/10.1101/2023.04.13.536745; this version posted August 29, 2023. The copyright holder for this preprint (which was not certified by peer review) is the author/funder, who has granted bioRxiv a license to display the preprint in perpetuity. It is made available under aCC-BY 4.0 International license.

Supplementary Figure Legends

Supplementary Figure 1. Cell-impermeable chelators effectively reduce Zn^{2+} levels in external media but do prevent initiation or continuation of Ca^{2+} oscillations.
(**A**) A representative trace of FluoZin3 fluorescence in replete monitoring media (TL-HEPES). The media was supplemented with cell-impermeable FluoZin-3, and after initiation of monitoring, the addition of EDTA (100 μM) occurred at the designated point (triangle). (**B**) The left black trace represents Ca^{2+} oscillations initiation by injection of *mPlcζ* mRNA (0.01 μg/μl). The oscillations were monitored in Ca^{2+} and Mg^{2+}-free media and in the presence of EDTA (110 μM) to chelate residual divalent cations derived from the water source or reagents used to make the media. The right red trace represents the initiation of oscillations as above, but after a period indicated by the black and green bars, Ca^{2+} and Mg^{2+} were sequentially added back.

Supplementary Figure 2. Overexpression of ER accessory protein ERp44 did not change the Ca^{2+} responses initiated by m*Plcζ* mRNA microinjection, Actylcholine, or $SrCl_2$.
(**A**) Representative immunofluorescent images of MII eggs with overexpression of ERp44. At 5 hr. post microinjection, eggs were treated with 10 or 50 μM of TPEN and incubated for 1 hr, after which they were fixed and stained. An anti-HA antibody was used. Scale bar: 10 μm. (**B**) Representative Ca^{2+} responses induced by m*Plcζ* mRNA microinjection (0.01 μg/ μl-left column), $SrCl_2$ (10 mM-center column), and acetylcholine (50 μM-right column) in eggs with (top panels) or without (bottom panels) ERp44 overexpression.

Supplementary Figure 3. Elevated Zn^{2+} impairs egg activation and the subsequent embryo development.
(**A**) MII eggs were incubated in TL-HEPES containing 0, 0.1, or 1.0 μM ZnPT at room temperature for 10 min and washed several times with fresh TL-HEPES and injected with m*Plcζ* mRNA. After it, eggs and zygotes were cultured in KSOM for 24h. PN formation and 2-cell development were checked at 7 and 24h post-microinjection. Bars represent the percentages of injected eggs that reached the PN and the 2-cell stage. Scale bar: 50 μm. (**B**) MII eggs injected with m*Plcζ* mRNA were incubated in KSOM without ZnPT for an hr. and then incubated in KSOM with 0 or 0.1 μM ZnPT for 24h. The second polar body extrusion, PN formation, and 2-cell development were checked at 2.5-, 7- and 24h. post-microinjection. Bars represent the percentages of injected eggs that reached the PN and the 2-cell stage. Scale bar: 50 μm.

Supplementary Figure 1.

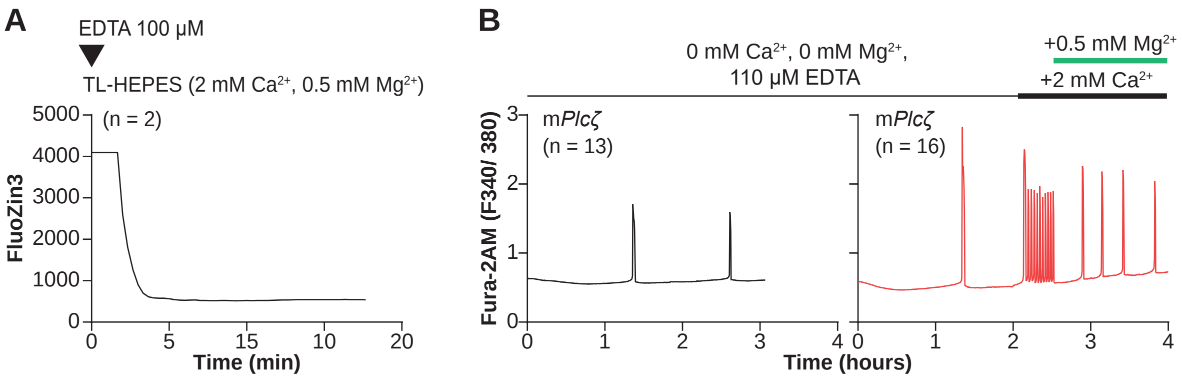

Supplementary Figure 2.

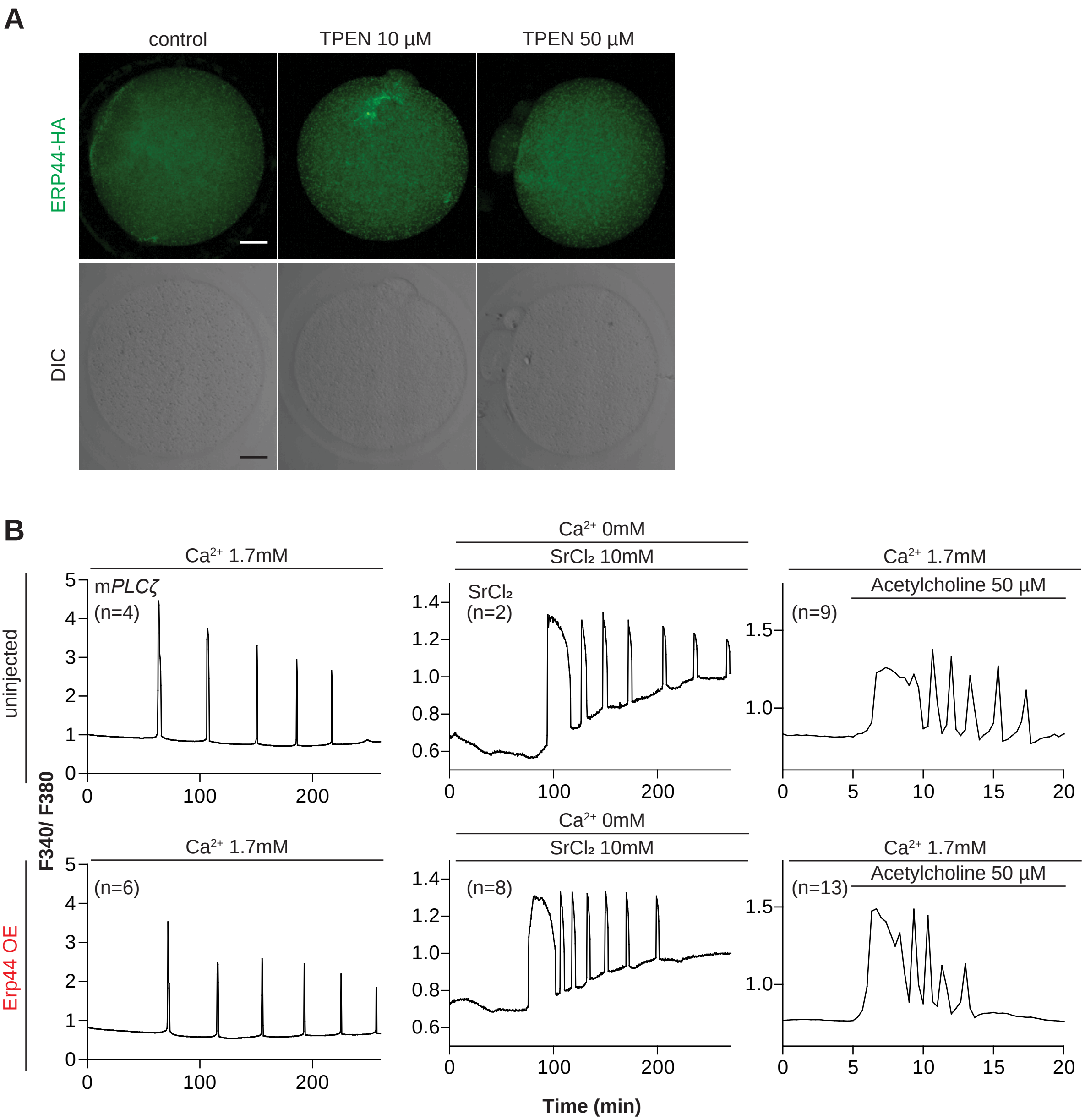

Supplementary Figure 3.

A

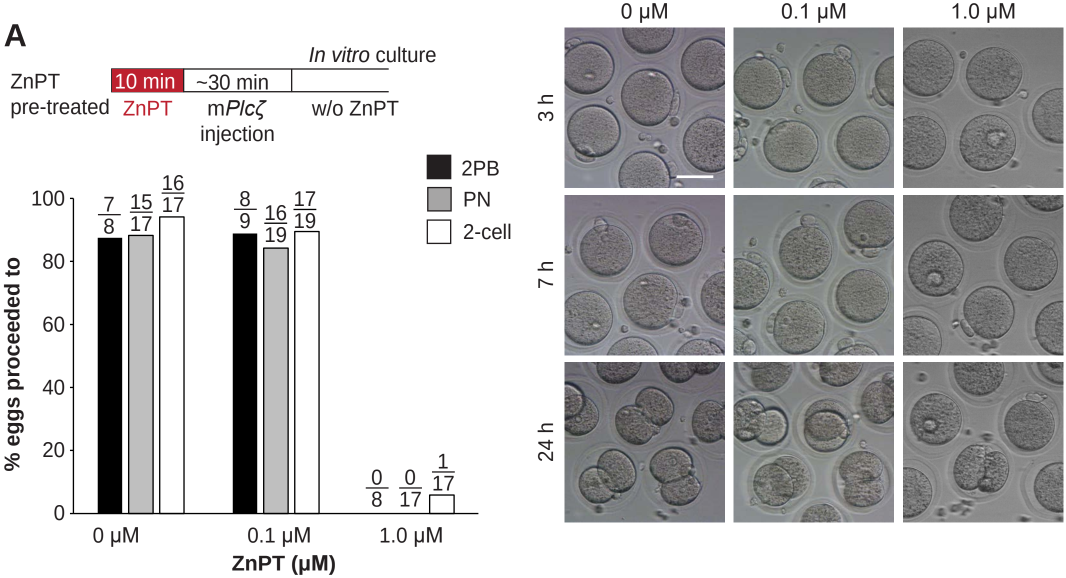

B

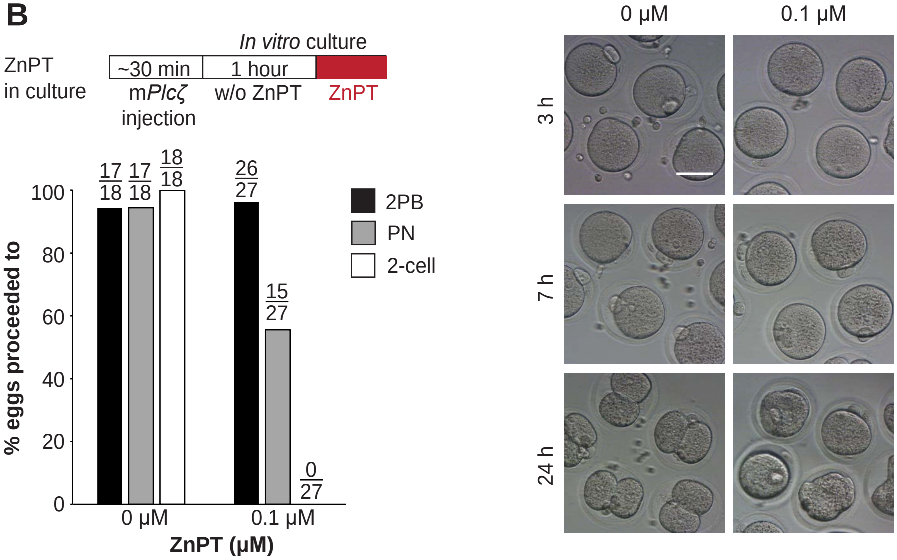

Supplement: Supplementary file 1 [file NIHPP2023.04.13.536745v3-supplement-1.pdf]
