# Supplementary material for: Zn2+ is Essential for Ca2+ Oscillations in Mouse Eggs
Source: bioRxiv. 2023 Apr 17:2023.04.13.536745. Preprint. [Version 2] doi: 10.1101/2023.04.13.536745 (PMC10153198; doi:10.1101/2023.04.13.536745)
Supplement: Supplementary file 1 [file NIHPP2023.04.13.536745v2-supplement-1.pdf]

bioRxiv preprint doi: https://doi.org/10.1101/2023.04.13.536745; this version posted April 17, 2023. The copyright holder for this preprint (which was not certified by peer review) is the author/funder, who has granted bioRxiv a license to display the preprint in perpetuity. It is made available under aCC-BY 4.0 International license.

Supplementary Figure 1. Overexpression of ER accessory protein ERp44 did not change the Ca^{2+} responses initiated by m*Plcζ* mRNA microinjection, Actylcholine, or $SrCl_2$.

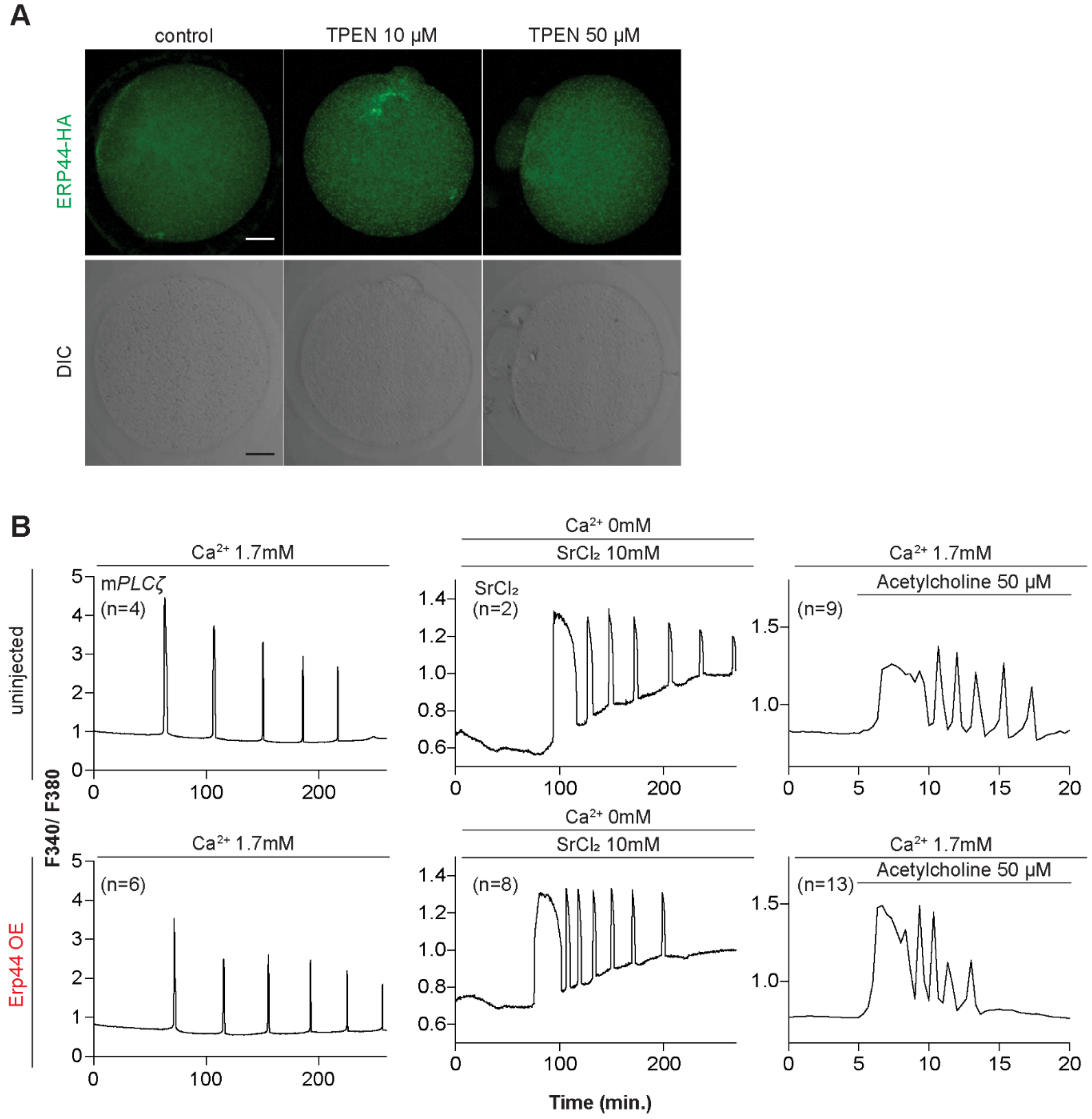

(**A**) Representative immunofluorescent images of MII eggs with overexpression of ERp44. At 5 hr. post microinjection, eggs were treated with 10 or 50 µM of TPEN and incubated for 1 hr, after which they were fixed and stained. An anti-HA antibody was used. Scale bar: 10 µm. (**B**) Representative Ca^{2+} responses induced by m*Plcζ* mRNA microinjection (0.01 µg/ µl-left column), $SrCl_2$ (10 mM-center column), and acetylcholine (50 µM-right column) in eggs with (top panels) or without (bottom panels) ERp44 overexpression.

bioRxiv preprint doi: https://doi.org/10.1101/2023.04.13.536745; this version posted April 17, 2023. The copyright holder for this preprint (which was not certified by peer review) is the author/funder, who has granted bioRxiv a license to display the preprint in perpetuity. It is made available under aCC-BY 4.0 International license.

Supplementary Figure 2. Elevated Zn^{2+} impairs egg activation and the subsequent embryo development.

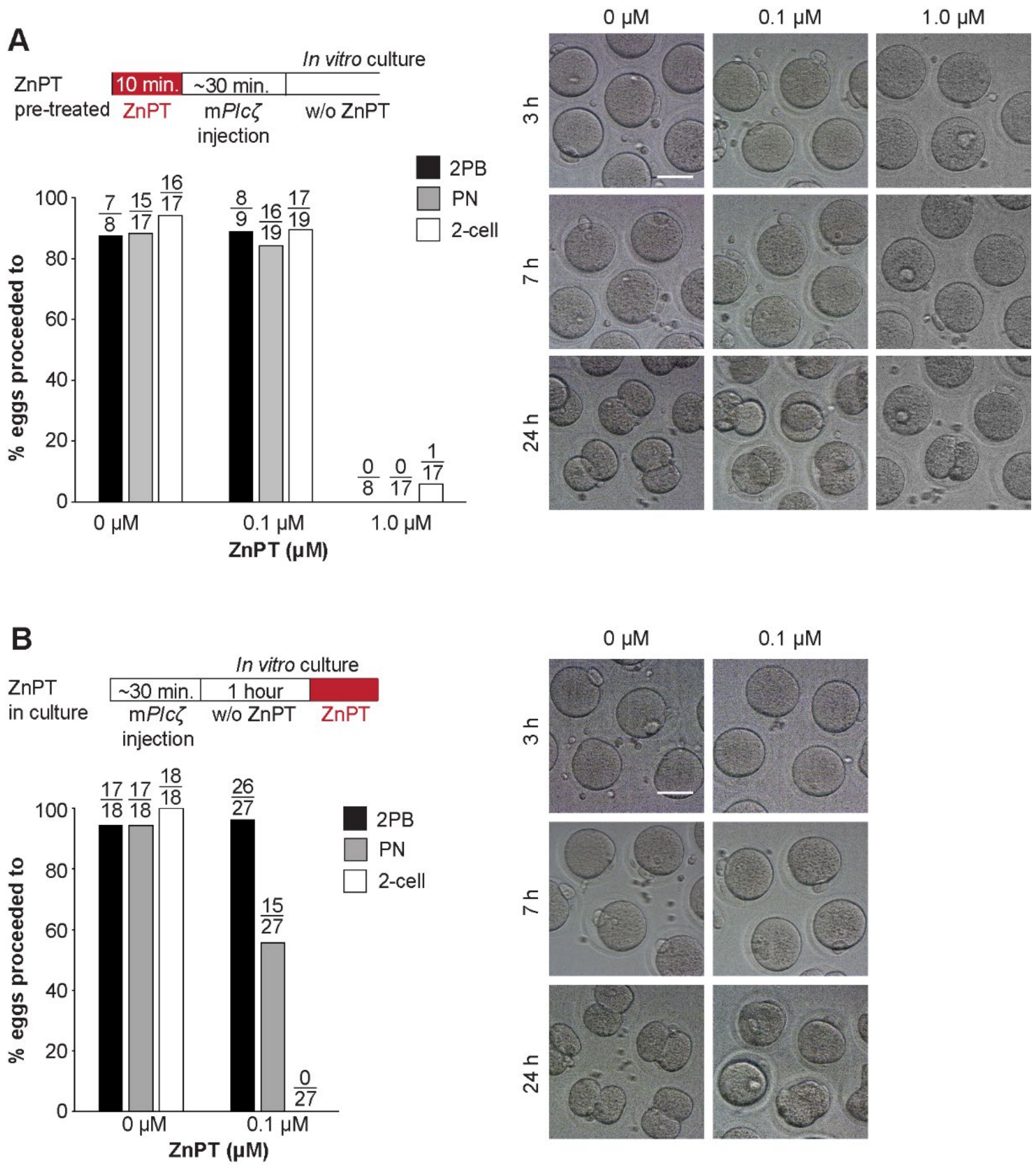

(**A**) MII eggs were incubated in TL-HEPES containing 0, 0.1, or 1.0 μM ZnPT at room temperature for 10 min and washed several times with fresh TL-HEPES and injected with m*Plcζ* mRNA. After it, eggs and zygotes were cultured in KSOM for 24 hr. PN formation and 2-cell development were checked at 7- and 24-hr post-microinjection. Bars represent the percentages of injected eggs that reached the PN and the 2-cell stage. Scale bar: 50 μm. (**B**) MII eggs injected with m*Plcζ* mRNA were incubated in KSOM without ZnPT for an hr. and then incubated in KSOM with 0 or 0.1 μM ZnPT for 24 hr. The second polar body extrusion, PN formation, and 2-cell development were checked at 2.5-, 7- and 24-hr. post-microinjection. Bars represent the percentages of injected eggs that reached the PN and the 2-cell stage. Scale bar: 50 μm.
